# Patient With Mild Alzheimer's Disease Homozygous for *ApoE* ε4 Showing Improved Cognition, No Brain Volume Loss, and Complete Amyloid Clearance After Lecanemab Treatment

**DOI:** 10.1111/ggi.70251

**Published:** 2025-11-14

**Authors:** Haruo Hanyu, Yumi Koyama, Toshimitsu Momose, Sadayoshi Watanabe

**Affiliations:** ^1^ Dementia Research Center Tokyo General Hospital Tokyo Japan; ^2^ Department of Geriatric Medicine Tokyo Medical University Tokyo Japan; ^3^ Department of Rehabilitation Tokyo General Hospital Tokyo Japan; ^4^ Department of Biofunctional Imaging Fukushima Medical School; ^5^ Department of Neurosurgery Tokyo General Hospital Tokyo Japan

Dear Editor,

1

Monoclonal antibodies targeting β‐amyloid (Aβ), including lecanemab and donanemab, have been approved for the treatment of mild dementia and mild cognitive impairment caused by Alzheimer's disease (AD). Although these antibodies reduce cognitive decline in AD patients, those who have the apolipoprotein E epsilon 4 (*ApoE* ε4) gene still show a high risk for amyloid‐related imaging abnormalities (ARIA), such as brain edema, microhemorrhages, and superficial siderosis [1]. In fact, lecanemab has not been approved for use in AD patients homozygous for *ApoE* ε4 in Great Britain, owing to an unfavorable risk–benefit ratio. In addition, subgroup analysis of a lecanemab trial demonstrated that patients homozygous for *ApoE* ε4 show less favorable treatment effects than *ApoE* ε4 noncarriers [2]. We herein report a case of a patient with mild AD who was homozygous for *ApoE* ε4, but showed improvement in cognition, no brain volume loss on magnetic resonance imaging (MRI), and complete amyloid clearance on positron emission tomography (PET) without any adverse events after 18 months of lecanemab treatment.

The patient was a 75‐year‐old man with 12 years of education. He had been receiving donepezil treatment from his family doctor for the past 3 years. He visited the memory clinic at our hospital to receive lecanemab treatment. His short‐term memory and orientation were impaired, and his Mini‐Mental State Examination (MMSE) score and the Japanese version of the Montreal Cognitive Assessment (MoCA) score were 22 and 19, respectively. The Clinical Dementia Rating (CDR) score was 0.5 (global) and 4.5 (sum of boxes [SB]). MRI (Siemens MAGNETOM Symphony, 1.5 T MRI system) displayed substantial medial temporal lobe atrophy, but no other abnormalities, except for one microbleed in the right occipital lobe, and PET (Discovery 610, GE Healthcare, ^18^F‐flutemetamol, 185 MBq) displayed amyloid positivity. Genetic testing demonstrated that his *ApoE* genotype was ε4/ε4. As he was diagnosed as having mild AD, and met the eligibility criteria for lecanemab treatment according to the Japanese guidelines for its optimal use, lecanemab treatment was initiated (10 mg/kg, biweekly). No infusion‐related reactions were observed. Follow‐up MRI at 2, 3, 6, 12, and 18 months displayed no ARIA or other adverse findings. Figure 1 shows the changes in cognition, MRI findings, and PET scans during lecanemab treatment. After 18 months of treatment, the patient's CDR‐SB score improved from 4.5 to 2.5, although his global CDR (from 0.5 to 0.5), MMSE (from 22 to 22), and MoCA (from 19 to 19) scores showed no changes. Lawton's instrumental activities of daily living score also improved from 2/5 to 3/5. Follow‐up MRI displayed no significant changes, and values of both the severity of volume of interest atrophy (Z‐score, from 3.28 to 3.13) and extent of gray matter (GM) atrophy (from 3.76% to 3.86%) measured using voxel‐based specific regional analysis system for AD (VSRAD) [3] were stable. A second PET scan after 18 months of lecanemab treatment demonstrated complete amyloid clearance (from 51.6 centiloids to 3.6 centiloids).

**FIGURE 1:**
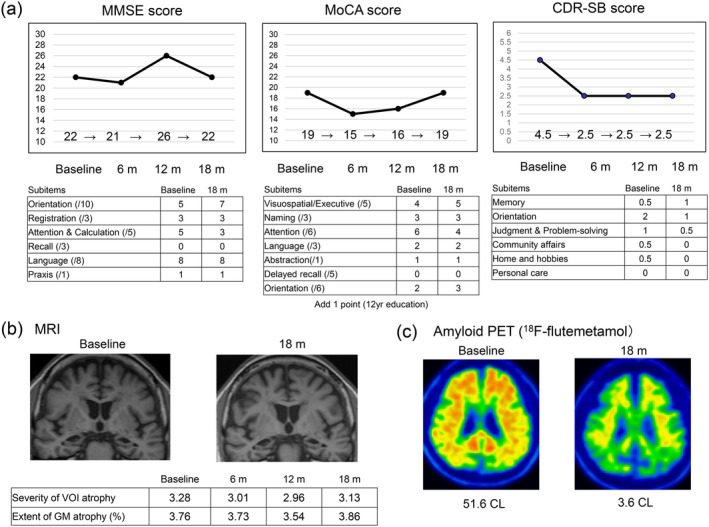
Changes in cognition, MRI findings, and PET scans between before and after lecanemab treatment. (a) Although MMSE and MoCA scores showed no changes from baseline at 18 months of lecanemab treatment, an improvement in CDR‐SB score was observed. (b) No significant changes on brain MRI between baseline and 18 months were observed, as values of both the severity of VOI atrophy and extent of GM atrophy analyzed using VSRAD were stable. (c) PET scans displayed complete amyloid clearance at 18 months. MMSE, Mini Mental State Examination; MoCA, Montreal Cognitive Assessment; CDR‐SB, Clinical Dementia Rating‐sum of boxes; MRI, magnetic resonance imaging; PET, positron emission tomography; VOI, volume of interest; GM, gray matter; CL, centiloid; m, months.

Compared with the phase 3 trial of lecanemab (Clarity AD), in which the mean CDR‐SB score change from baseline to 18 months was 1.21 in the lecanemab group vs. 1.66 in the placebo group [1], the present patient showed an improvement in CDR‐SB score (−2.0 change). Follow‐up assessment using comprehensive neuropsychological tests is needed. Although lecanemab mainly binds with high affinity to soluble Aβ protofibrils, the complete clearance of amyloid deposition was observed in the present patient (93% reduction of amyloid) on PET at 18 months. Moreover, no significant brain volume loss was observed. Although accelerated brain volume loss was shown in some recent studies on amyloid‐removing therapies [4], an overall association between the treatment effects on brain atrophy and clinical outcomes has been reported [5]. Although the characteristic features and backgrounds of patients who respond positively to lecanemab remain unknown at present, the amyloid burden at baseline of our present patient was not very high (51.6 centiloids). Therefore, as patients in the low amyloid subgroup (< 60 centiloids) showed continued benefit in a long‐term lecanemab trial [6], patients with low amyloid accumulation, indicating the earlier stages of AD, may show greater benefit than those with high amyloid accumulation. Further research is needed to elucidate the underlying mechanism of the effects of lecanemab, to provide safer and more effective therapies for patients. Although anti‐amyloid therapies for patients who are *ApoE* ε4 carriers must be carefully considered owing to the higher risk of ARIA, lecanemab treatment may be an option even for homozygous *ApoE* ε4 patients, as our present *ApoE* ε4 homozygous patient showed a favorable response to lecanemab.

## Disclosure

The authors have nothing to report.

## Consent

Written consent was obtained from the patient for publication of this article.

## Data Availability

The data that support the findings of this study are available on request from the corresponding author. The data are not publicly available due to privacy or ethical restrictions.
